# “She’s a dog at the end of the day”: Guide dog owners’ perspectives on the behaviour of their guide dog

**DOI:** 10.1371/journal.pone.0176018

**Published:** 2017-04-19

**Authors:** Peter J. Craigon, Pru Hobson- West, Gary C. W. England, Chantelle Whelan, Emma Lethbridge, Lucy Asher

**Affiliations:** 1 School of Veterinary Science and Medicine, The University of Nottingham, Leicestershire, United Kingdom; 2 Guide Dogs for the Blind Association, Burghfield Common, Reading, United Kingdom; 3 Centre for Behaviour and Evolution, Henry Wellcome Building, Newcastle University, Newcastle, United Kingdom; University of Missouri Columbia, UNITED STATES

## Abstract

A guide dog is a domestic dog (*Canis familiaris*) that is specifically educated to provide mobility support to a blind or visually impaired owner. Current dog suitability assessments focus on behavioural traits, including: trainability, reactivity or attention to environmental stimuli, low aggressiveness, fearfulness and stress behaviour, energy levels, and attachment behaviour. The aim of this study was to find out which aspects of guide dog behaviour are of key importance to guide dog owners themselves. Sixty-three semi-structured interview surveys were carried out with guide dog owners. Topics included the behaviour of their guide dog both within and outside their working role, and also focused on examples of behaviour which might be considered outside a guide dog owner’s typical expectations. Both positive and negative examples and situations were covered. This allowed for the discovery of new perspectives and emerging themes on living and working with a guide dog. Thematic analysis of the results reveals that a dog’s safe behaviour in the face of traffic was the most important positive aspect of a guide dog’s behaviour and pulling or high tension on the lead and /or harness was the most discussed negative aspect. Other aspects of guide dog behaviour were highlighted as particularly pleasing or disappointing by owners including attentiveness to the task, work, environment and owner; confidence in work and decision making (with confident dogs resulting in confident owners) obedience and control; calmness and locating objectives. The results reveal important areas of behaviour that are not currently considered priorities in guide dog assessments; these key areas were consistency of behaviour, the dog’s maturity and the dog’s behaviour in relation to children. The survey revealed a large range in what owners considered problematic or pleasing behaviours and this highlights the heterogeneity in guide dog owners and the potential multifarious roles of the guide dog. This study contributes to the literature on which behaviour is considered appropriate or inappropriate in dogs and on the nature of human-animal interactions.

## Introduction

A guide dog is a domestic dog (*Canis familiaris*) that is specially trained to provide mobility support to a blind or visually impaired owner. As a mobility aid guide dogs can improve an individual’s physical activity level, distance travelled, the pace of walking speed, and familiarity of routes considered [1,2,3,4,5]. Beyond their trained role in mobility support, guide dogs can also provide: feelings of increased independence, confidence and safety[2,3,6,7]; affection and companionship[2,3]; facilitation of social interaction[2,5,6,8]; and positive changes in social identity[8]. Published literature suggests that the majority of guide dog owners assert that having a guide dog has had a positive impact on their life[2,3,6], despite the associated inconveniences and limitations. These limitations might include, but are not limited to, the responsibility of caring for a dog, limitations of places where it is possible to take a dog, behavioural issues, soreness of muscles or pull on one side of the body, receiving unwanted attention, or feelings of loss and bereavement at the end of a working partnership[4,5].

A central aspect of the guiding role is that a dog in harness should continue in a given direction as directed by the handler, negotiating in a safe way any hazards or obstacles that may be encountered. Dogs are also trained to follow obedience and directional commands, walk ahead of a handler when in harness with some tension but not pulling, approach and sit at kerbs, stop to avoid an approaching vehicle and locate certain objectives. Training many of these working tasks to a consistent level requires significant time and input. As a result much research has focused on developing assessment methods, which can predict success in training. For example, by conducting a series of behavioural tests on potential working dogs during training, Svartberg (2002) found the level of boldness in the dogs was predictive of subsequent working success[9].

Assessments of puppies and juvenile dogs have been carried out to predict chances of success as guide dogs[10,11]. Questionnaires administered to handlers or volunteer caregivers with knowledge of a dog in training have also been used to assess suitability for guide dog training[12,13,14]. Such assessments focus on behavioural suitability for the future role and highlight suitable levels of the following behaviour as being important for guide dogs: responsiveness to or cooperation with a handler (trainability), reactivity or attention to environmental stimuli (often referred to by guide dog organisations as distractions or suspicions), low aggressiveness, fearfulness and stress behaviour, not having high energy levels, and showing affiliate or attachment behaviour[10,13,14,15,16,17,18]. Studies that consider behaviour of working guide dogs are less common (although see Caron-Lormier et al. in press for an exception)[19].

In addition to the guiding role of guide dogs, they can also fulfil the role of companion and have been described as working pets[20]. For owners of companion dogs, a dog’s appearance and perceived personality are important factors when selecting a dog[21,22]. Behaviours deemed undesirable by owners include: soiling in the house, damaging or scratching the furniture, excessive vocalising, boisterousness, aggression towards animals or people and separation or fear related behaviour[23]. Perhaps the most important aspect of a pet dog is the attachment the owner feels towards it. This appears to act as a buffer for relinquishment, even when dogs are showing undesirable behaviour.

Guide dog owners range from young, active people with busy schedules to elderly individuals who may only need guiding by their dog once or twice per week on a limited number of routes. Guide dog owners also vary in factors such as the level of vision, domestic circumstances, culture, and the area in which they live (e.g. urban or rural). Demographic and contextual factors are known to influence the reasons for applying for a guide dog and the perceived advantages and disadvantages of guide dog ownership[5].

Currently, research on guide dog behaviour typically considers the dog’s behaviour from the perspective of standardised canine suitability assessments. The novel aim of this study was to find out which aspects of guide dog behaviour are of key importance to the guide dog owners themselves in the context of their day to day experience of living and working with a guide dog. This is an element that cannot be captured in a standardised assessment and will help inform the use of such assessments and training of guide dogs, through providing insight and giving context as to how a dog’s behaviour manifests itself in its life with an owner and how the owner views this. There was no specific hypothesis as this project was intended to explore guide dog owners’ views of the behaviour of their dogs. It was aimed that an in depth understanding of the range of the behaviour of guide dogs in real life working situations would emerge along with a nuanced appreciation of the varied affects that this has on their owner and work as a partnership.

## Methods

### Procedure

The researchers were provided with a list of contact details for guide dog owners (GDOs) from the Guide Dogs for the Blind Association UK (henceforth Guide Dogs) who had previously indicated that they were willing to be contacted to participate in research studies. The owners were contacted in random order (by randomising the order of the list of contact details) until saturation was reached. This was decided in terms of having covered: experience of Guide Dog ownership (number of Guide Dogs owned), demographic factors such as age and gender and working requirements of the dog; (eg high/ low workload and urban or rural environments). It was also verified that the GDOs currently had a working Guide Dog, so GDOs that were between dogs on the waiting list were ineligible. After this initial phone call introducing the survey, a convenient appointment was made with the primary author (PC) within the next week to complete the telephone survey. During this initial call, informed consent was obtained by reading a script which had ethical approval from the University of Nottingham. The option was provided to receive a copy of the informed consent details in preferred format (e.g. large print or braille, email or post). No GDOs declined to take part in the study but two owners were not contactable at their arranged time after multiple attempts, so did not participate in the study. It was made clear that all questions were optional and GDOs did not have to answer a question if they did not wish to although this did not occur.

Participant’s responses were recorded near verbatim by note taking during the survey. These notes were compiled into a standardised pro-forma structured around the questions given below and then transferred into Microsoft Excel. Where prompting for extra explanation or information was used, or where responses were missed, this was marked. Responses were checked with the GDO during and at the end of the interview to ensure accuracy. The responses were then analysed following the procedure given below.

### Survey

The telephone survey consisted of 39 questions (see Table 1) covering: demographics (16 questions); guide dog work (5 questions); examples of dog behaviour (8 questions); score of overall dog behaviour (1 question) score of specific dog behaviour with requests for elaboration (9 questions). These questions were decided via discussion with the research team and experts within Guide Dogs to cover all behavioural situations (see below). The questions were piloted with 5 local GDOs over the telephone, which demonstrated their appropriateness and efficacy at eliciting the desired conversational account of their dog’s behaviour.

**Table 1:** GDO survey questions–in order that they were asked.

**Demographics—History of Visual Impairment and Contact with Guide Dogs** **Are you registered as; blind, partially sighted or not registered?** **For roughly how many years have you had a visual impairment that you would say has affected your day-to-day life in some way? (If you have had this since birth, please say so).** **How long ago were you first trained with a guide dog?** **What age were you when you first became a guide dog owner?How many guide dogs (including all the dogs you have qualified with) have you owned in total? (NB- ‘All the dogs that you have qualified with’ was included to capture all of the guide dogs the GDOs had owned. This would ensure the inclusion of guide dogs that the GDO may have qualified with but owned for such a short length of time that they did not remember them as a successful partnership or work with them for a significant length of time.)**
**Guide Dog work section** **What breed and is your current guide dog? Is it male or female?** **How long have you had your current guide dog?** **How old in years is your current guide dog?** **How many times per week does your dog guide you in harness?** **How many routes do you use with your current guide dog? Where are these?** **How satisfied on a scale of 0 to 100 where 0 is not at all satisfied and 100 is completely satisfied are you with the behaviour of your current guide dog?**
**GDO—specific scenario questions** **Positive** **Can you explain any particular situation when your dog’s behaviour exceeded you expectations?** **What was the situation?** **What did the dog do that was so good?** **To what extent did this affect your work and relationship with the dog?** **Negative** **Can you explain a particular situation when your dog’s behaviour disappointed you?** **What was the situation?** **What did the dog do that was so disappointing?** **To what extent did this affect your work and relationship with the dog?**
**Specific Scenario Questions** **In Harness** **On a scale of 0 to 100 where 0 is awful and 100 is perfect please rate your dog’s behaviour when it is with you in harness. Can you please explain your response.** **What, if anything, would you like your dog to do differently in these situations?** **To what extent does this affects your work and relationship with the dog?** **On a lead** **On a scale of 0 to 100 where 0 is awful and 100 is perfect please rate your dog’s behaviour when it is on a lead and out of harness. Can you please explain your response.** **What, if anything, would you like your dog to do differently in these situations?** **To what extent does this affect your work and relationship with the dog?** **Neither in harness nor on a lead** **On a scale of 0 to 100 where 0 is awful and 100 is perfect please rate your dog’s behaviour when it is neither in harness nor on a lead. Can you please explain your response.** **What, if anything, would you like your dog to do differently in these situations?** **To what extent does this affects your work and relationship with the dog?**
**Demographics—continued** **Are you male or female?** **What is your exact age?** **What is your current marital status?** **What is your current employment status?** **Which ethnic group do you consider yourself to belong to?** **In which regions do you live?** **How would you describe the place where you live? E.g. village, town, city centre** **Do you have any other pets or animals in the house apart from your guide dog?** **How many people live in your household including yourself?** **How many of these people are under 18?** **What age are these children?**

A semi-structured interview approach with a mixture of open and closed questions was employed in order to ensure coverage of the desired range of behavioural situations (see below). This was intended to allow for a degree of comparability between participants. The survey was conducted via telephone rather than face-to-face. This was for reasons of practicality (time, cost and convenience to the GDO and researcher) and the need to access participants who were distributed across the UK. This was particularly appropriate for participants who by their nature were visually impaired. The desire to elicit a conversational account of the guide dog’s behaviour meant that a telephone survey was chosen over written methods and the importance of individual accounts meant that it was conducted on a one to one basis rather than via a focus group for example.

To elicit a conversational account of the participant’s views on the behaviour of their dog, a mixture of open ended and closed questions were used throughout. GDOs were asked to give examples of situations where their dog’s behaviour was positive and then negative and how this situation had affected the work and relationship with the dog. GDOs were asked to provide a score of their satisfaction with their dog’s behaviour, overall and then when in harness, lead and neither in harness or on a lead. This distinction was to encourage participants to consider working (which happens primarily in harness) as well as non-working situations to allow a fuller understanding of the GDO-dog relationship.

A score was provided by the GDO for 12 questions on a scale of 0 to 100, where 0 was indicated to be ‘awful’ and 100 ‘perfect’ according to the GDO’s understanding. This indicated the GDO’s satisfaction with their dog’s behaviour in harness, when on lead and when neither on lead or in harness. Participants were asked to explain their response, what they would have liked the dog to do differently, and how this affected their work and relationship with the dog. The scores were used to prompt discussion about the different aspects of dog behaviour and to provide context for the impact of these behaviours on the GDO. They were not considered as a quantitative ‘measure’ and were therefore not analysed as such.

The purpose of this research was to elicit information from GDOs about the behaviour of their dog, through an informal chat. In order to secure the required contact details for the GDOs from Guide Dogs the research was framed in this context. It was therefore decided at a very early stage that the interviews would not be audio recorded but captured by note-taking. Whilst it is acknowledged that this potentially means important information was missed, it was felt that audio-recording the interviews would have added an extra level of formality that may have discouraged participation and changed the responses of the participants. Permission for this research was negotiated on these terms and the research was designed with this in mind.

Recording the interviews through note taking influenced the design of the survey in the following ways: The survey was made to be short and semi-structured taking roughly twenty minutes to complete and was recorded using a structured proforma. This brevity and structure improved the accuracy of the recording of responses. The survey was focused on eliciting descriptive accounts of behaviour from the GDOs not their use of language or other contextual information, therefore allowing the researcher to concentrate on recording these descriptive accounts. A greater number of responses was also sought to mitigate any effect of recording the responses through note- taking. This approach was also mitigated through all of the responses being recorded by a single researcher, ensuring a consistent approach to recording and analysis, validated with discussion with other experts and team members as described below.

### Qualitative analysis

Framework analysis was carried out according to the method described by Ritchie and Spencer (1994)[24]. A thematic framework derived from Guide Dogs’ existing behavioural assessment framework and behavioural withdrawal reasons (behavioural reasons for a dog being withdrawn from training or work) comprising 19 themes and 5 overarching categories was used for analysis (see Table 2). This was applied to each individual participant’s responses before amalgamating the data for each theme and finally interpreting the data within these themes.

**Table 2:** Framework (categories and themes) used for analysis of GDOs responses and aspect of Guide Dogs’ assessment this was based on.

Category	Theme	Definition	Guide Dogs training assessment and withdrawal reasons covered by this theme
Attraction towards people, animals or objects	1 Attentiveness	Focus of the dog on a person or task.	Includes assessment of ‘Attentiveness’ during training and withdrawal reasons ‘Attentiveness–Low–Handler focus’ & ‘Attentiveness–Low–Task Focus’.
	2 Distraction	Attraction to items in the environment.	Includes assessment of ‘Distraction’ during training and withdrawal reasons ‘Distraction for: Objects/Food/People/ Animals or Birds/ Sounds/Scents/ General’.
Responses to people animals or objects in the environment	3 Confidence	Choosing a course of action with little human intervention.	Includes assessment of ‘Confidence’ during training and withdrawal reason ‘Confidence–Low Adaptability’.
	4 Stress Resilience–Anxiety Degree	Indications of stress shown across a variety of situations.	Includes assessment of ‘Stress Resilience–Anxiety Degree’ during training and withdrawal reasons ‘Stress Resilience–Low’.
	5 Suspicion–Anxiety Degree	Fear or stress associated with specific stimuli.	Includes assessment of ‘Suspicion–Anxiety Level’ during training and withdrawal reasons ‘Suspicion High to Objects/People/Animals/Sounds/Scents/ General’.
	6 Aggression	Shows signs of agonistic behaviour.	Includes assessment of ‘Aggression People/Animals’ during training and withdrawal reasons ‘Aggression People/Animals’.
	7 Interaction People and 8 Interaction Animals	Displaying desired (excluding nonaggressive) behaviour when in contact with a person or animal.	Includes assessment of ‘Interaction People’ and ‘Interaction Animals’ during training.
Training	9 Eager/ Willing	Motivated to perform guiding and other tasks.	Includes assessment of ‘Eagerness/ Willingness’ during training.
	10 Obedience	Responsiveness to commands.	Includes assessment of ‘Obedience’ during training.
	11 Skills / Task Acquisition	Leading a person away from obstacles along a logical path.	Includes assessment of ‘Straight Line Work’, ‘Kerb Work’, ‘Right Shoulder Work’ and ‘On Kerb Off Kerb Work’.
	12 Locating Objectives	Guiding towards a specific named object.	Includes assessment of ‘Locating Objectives’ during training.
	13 Traffic	Not guiding across roads when traffic is approaching.	Includes assessment of ‘Traffic’ during training.
	14 Calmness	Not being easily agitated.	Includes assessment of ‘Calmness’ during training and withdrawal reasons ‘Social Behaviour–Hyperactivity/ Boisterous’.
Non-guiding behaviour	15 Inappropriate non-working behaviour	Displaying behaviour that is generally agreed to be unwanted.	Includes assessment of ‘Behaviour When Left’, ‘Social Behaviour–Noisy When Left’, ‘Destructive’, ‘Scavenge–Scrounger’ during training and withdrawal reasons ‘Social Behaviour–Noisy When Left/ Destructive’ & ‘Social Behaviour/Scavenge–Scrounger’.
	16 Behaviour on Transport	Guiding, locating objectives and calm behaviour on modes of transport.	Includes assessment of ‘Behaviour on Transport’ during training.
	17 Toileting (spending) and Coprophagia	Defecation at appropriate times and places (e.g. when provided the opportunity in a specific area, rather than when guiding) and consumption of faecal matter.	Includes assessment of ‘Spending’ (toileting) during training and withdrawal reasons ‘Social behaviour (Toilets Indoors)’ and ‘Social Behaviour—Coprophagia’.
Behaviour related to lead or harness	18 Body Sensitivity	Showing a negative reaction to harness, collar, lead or touch.	Includes assessment of ‘Body Sensitivity’ during training and withdrawal reason ‘Body Sensitivity–High’.
	19 Position and speed when in harness or on lead	Maintaining a parallel position of regular speed slightly ahead of the handler when in harness and walking to heel when on the lead.	Includes assessment of ‘Handler Position in Quiet and Busy Areas’ and ‘Speed Control’ during training.

To achieve this, each of the responses was repeatedly re-read with each of the categories in mind and responses were colour coded according to the relevant category. Once this process was completed, comments for each theme were extracted and grouped together to provide the perspectives under each category theme to allow for the assessment of priority and relative importance to the GDOs. There were areas which did not fit into any of these themes or categories and these gave rise to emergent themes identified inductively from participants’ responses. During this process, attention was paid to important comments that did not fall into any of the pre- existing categories with these being grouped to form the emergent categories of particular importance to GDOs.

The researcher identified the relative importance of themes by following broad guiding principles including: the frequency with which an issue was mentioned, the language used to describe the behaviour and whether the GDOs indicated that the effect was significant in terms of disrupting or improving their work, routine, confidence or feelings when with the dog. The satisfaction score given for the dog’s behaviour in certain scenarios was also used for this purpose. In order to provide greater context and understanding of the dog’s behaviour, the responses from the GDOs were also compared with qualitative data collected about the dog during raising and training and held centrally on a Guide Dogs’ database. This consisted of qualitative ‘free-text’ summaries recorded monthly by trainers during raising and training.

To ensure consistency, all of the coding and analysis of the responses was conducted by the same researcher. This was not done in isolation however as the analysis was subject to repeated presentation to, and discussion with, other members of the project team and experts from Guide Dogs more widely. This validated the results according to the extensive expertise and experience of those responsible for training guide dogs for their GDOs.

This research was undertaken as part of a project funded by Guide Dogs for the Blind UK. The data collected through these interviews is therefore the property of Guide Dogs for the Blind UK. The data contained within this paper has been approved for publication by Guide Dogs and requests for further data from the project can be made to Guide Dogs by contacting Helen Whiteside on helen.whiteside@guidedogs.org.uk.

## Results

### Participants

Sixty-three participants took part in this study. 30 participants were female and 33 male. They ranged in age from 22 to 98 years of age with a median age of 57. They had had their current guide dog for between 5 days and 9 years (108 months) with a mean of 31 months and standard deviation of 23 months. The dogs were worked on between 1 and 55 individual routes with the median being 5. Their current dog was between the 1st and 9th guide dog that they GDO had owned with the median number of dogs owned being 2. The current dog was the first dog for 30 GDOs.

### Survey responses

GDO’s responses did not map exactly onto Guide Dogs’ behavioural categories. Certain Guide Dogs’ categories were therefore combined or omitted from this analysis as they were not mentioned by the GDOs or were of little significance as indicated in Table 3. This explains the discrepancy between the number of categories listed in Table 2 above and those discussed during these results as indicated in Table 3. The thematic categories used are given in Table 2. Each theme is then discussed in turn below, with indicative quotes provided or context briefly explained.

**Table 3:** Overview of comments made by GDOs within each theme.

Framework Themes	Overview of comments in this theme
1. Attentiveness	Attentive dogs would show an immediate response to GDOs, but others showed low attentiveness by pre-empting instructions. Sometimes dogs would ‘switch off’, but GDOs also said that their dog would ‘Know who’s boss’. Attentiveness would vary by situation, particularly when in and out of harness. Sometimes dogs would be attentive through proximity-seeking and some were described as over-attentive. Dogs were praised for being attentive to other people and also attentive in exceptional circumstances.
2. Distraction	Things that distracted dogs included: Food, dogs, people, scents, teddy bears, and other animals. Dogs were praised for not being distracted. Distraction had physical effects, caused accidents, and feelings of anxiety and trepidation.
3. Confidence	Confidence allowed GDOs to rely on the dog to do their job, resulting in feelings of safety and mutual confidence. Some GDOs wanted control and commented about over-confidence. Others mentioned their dog’s lack of self-confidence, which required work to build confidence as a partnership.
4. Stress Resilience	GDOs described their dogs as coping with a lot and stressful situations. Some dogs were described as nervous or skittish, and having good days and bad days. Stress resilience resulted in a bond, and attachment between dog and GDO.
5. Suspicion	Some dogs were suspicious of other dogs, and others required support from their GDO in certain situations. Suspicion was in some cases beneficial and dogs were praised for not being frightened. *Insufficient Comment*
6. Aggression	Some GDOs reported behaviour that could be linked to aggression including: Barking, growling and nipping at hands. GDOs also commented how their dogs would not be provoked and were not aggressive in the face of aggression.
7. Interaction people	GDOs praised their dogs for being good with children. Others mentioned how their dog was overfriendly or attention seeking or excited requiring control when with people.
8. Interaction animals	GDOs talked about their dog’s interaction with cats and other pets. Sometimes they were excited or nervous or would ignore the GDOS or be difficult to recall. GDOs also described how dogs behaved when attacked.
9. Eager/Willing	Dogs were described as enjoying their work, some exhibited low willingness and others were described as trying their best. Some dogs switched on and off, and GDOs mentioned how their dog’s eagerness matched to the GDO’s required level of work.
10. Obedience	GDOs talked about how their dog would or would not listen and respond. GDOs discussed good or, poor recall. Some dogs would look for instructions, whereas others would need them repeating, Some described it as a process of ‘give and take’ that varied in and out of harness. Obedience strengthened the bond with the dog and prevented problems from occurring.
11. Skills Acquisition	‘Straight Line Work’, ‘On Kerb Off Kerb Obstacles’, ‘Right Shoulder Work’, ‘Kerb Work’ (NB These are the technical elements of the guiding role that were less apparent to GDOs)–*Insufficient Comment*
12. Locating objectives	For some dogs one instruction was enough for them to find an objective with GDOs commenting on their dog’s problem solving. Dogs would recognise significant objects such as bins or shop counters. A dog’s ability to find objectives would boost the GDOs’ confidence, trust and reliance, which were affected when their dog missed objectives.
13. Traffic	Safety in traffic was the most significant single issue for GDOs. The GDOs’ confidence was affected when their dog was inconsistent in traffic.
14. Calmness	GDOs praised calm dogs. Dogs were excited: generally, by people, by novelty, and particularly when on lead or going for a free run. Excitability sometimes had an unsettling impact on the GDO, but others had a positive view of their dog’s excitement.
15. Inappropriate non-working Behaviour	Inappropriate behaviour included: stealing, destruction, and scavenging, GDOs reacted in different ways, or gave explanations for their dog’s behaviour. Barking and vocalisation was both seen positively and negatively with GDOs, accepting and giving excuses for ‘inappropriate’ behaviour.
16. Behaviour on transport	Occasionally GDOs said their dog was good on transport or showed initiative–*Insufficient Comment*
17. Toileting (Spending)	Some dogs would toilet in inappropriate places, times, or inconsistently. The effects varied with some GDOs ‘able to cope’ or ‘get over it.’ Some dogs would toilet in the house or be coprophagic.
18. Body sensitivity	Issues with the harness. *Insufficient Comment*.
19. Position and speed when in harness or on lead	GDOs commented on there being too much or not enough pace or tension on the harness, and praised ideal pace or tension on the harness. Dogs were praised for walking to heel on the lead but also often pulled on the lead. Dogs also learned to adjust their position relative to the handler, GDO or other person handling the dog.

The focus of the interviews was on behaviour, but in addition certain overarching themes emerged which relate to the relationship of the GDO with the dog and the emotional affect of the dog’s behaviour on the GDO. Frequent references to themes of trust, confidence, pride and mutual reliance, were used to indicate both the significance of behaviour and the role of a guide dog in the lives of their GDOs. This was a particular focus of the GDO’s comments and emphasises the importance of social behaviour and the ‘pet’ aspect of a guide dog as the time working in harness is often comparatively small in the relationship between GDO and dog. This importance of the GDO’s interaction and behaviour is considered further in the discussion.

#### Attentiveness

The level of attentiveness which pleased GDOs was when dogs needed only one clear command to follow a route or locate litter bins or counters in shops (even in places they had not been to previously) as when going to the bank as shown below.

“*If I say bank today*, *he’ll take me straight there*.*”*

When in harness a dog was often described as being in ‘work mode’ focusing on its job. Again dogs which were unpredictable in attentiveness caused trepidation and anxiety. Attentiveness was mentioned in connection with dog-GDO trust and bond.

“*It makes you two sides of the same coin, you get into each other's minds, there's a connection between you*”

A very high level of GDO-focused behaviour out of harness was considered as positive by some GDOs but negative by others.

“*He won't go too far away from me (he's) always looking around making sure I don't get into trouble*”

“*If I move away from where I work she witters. It's not feasible for me to be with her 24/7… (It's) like a child searching for reassurance. (I'd like her to) chill out with being left for 2–3 minutes (so it) wouldn't be a problem*.”

#### Distraction

Nearly every GDO mentioned that their dog had an interest that could develop into a problematic distraction depending on their obedience, attentiveness and relationship with their GDO. The range of things which distracted a guide dog from its work fall into the categories of food, other dogs, people, scents and other animals. The effect that distractions had on a dog’s work ranged from almost insignificant to major disruption. Distraction behaviour often began as a pull on the harness.

“*She can be a bit dog distracted, (she'll) pull wag and quicken her pace. (It) was a worry at first, (she) may take me in the wrong direction, now I can control it with my voice*.”

Often the dog was not blamed for distractions as other people were considered responsible for distracting the dog.

“*People are more of a distraction, people pat him, touch him. People are a pain, if you left him to work there wouldn't be a problem. I'm the only GDO in the area so I’m a bit of a novelty. People want to touch him*.”

The major effects of a dog’s potential for distraction were feelings of uncertainty and trepidation because distractions made the dog’s behaviour unpredictable.

“*If I'm going out I don't know how he's going to behave*.”

#### Confidence

GDOs summed up confident dogs by saying that they could rely on them to think for themselves and to do their job successfully. Confident decision-making strengthened the relationship with the dog. A key area for GDOs was when the dog correctly ignored GDO commands which kept the dog and GDO safe. Confidence was particularly important with regards to traffic, which was mentioned often. However, overconfidence was considered negative by the GDOs, for example, making navigational decisions without heeding GDO commands.

“…*he takes it upon himself to know where we're going, I'd rather he didn't*.”

A lack of confidence was discussed in negative terms, although some GDOs acknowledged that this was understandable in certain circumstances. Some GDOs described their dogs as requiring support or direction in both working and non-working situations and felt this required more input from them to prevent mistakes.

#### Stress resilience

Stress or anxiety were words rarely used by GDOs, however dogs were described as nervous or skittish in certain circumstances. The focus of the GDO’s comments was on praising how well their dogs coped with the varied demands of their work, extending to situations which the GDO themselves found stressful.

“*I have 3 children (and she) tolerates a variety of things. What she has to cope with (from) being poked and prodded to coping with crowds in London*”

#### Interactions with people and animals

A predominant, emergent theme with regard to interactions with people was GDOs’ pleasure at how well their dogs behaved in relation to children and other animals.

“*I've got a small granddaughter and she's wonderful with her. (She) could touch her food*.”

The friendliness of their dogs was viewed positively by most GDOs, but was occasionally considered to invoke unwanted attention and distraction, often attributed to overexcitement or immaturity.

A couple of GDOs recounted times that they and their dog had been attacked by other dogs and praised their dog’s calm or protective behaviour in the face of this aggression

#### Eagerness and willingness

Many of the GDOs commented that their dog enjoyed their work, which was reflected by them waiting to work and putting a lot of effort into their work.

“*(she's) always keen to work, excited (and) waiting to work*”

However, this attitude to work needed to be appropriate to each GDO, with comments being made that their dogs were happy with either a lot or a little to do. Dogs were also indicated to very occasionally lack willingness at certain times and under certain circumstances. These instances were recounted as isolated incidents, but caused uncertainty in the GDOs until they had regained the confidence that their dog would not do it again.

#### Obedience

Obedience was of great importance to GDOs, contributing greatly to a positive relationship with their dog. Dogs were praised when they did what they were told, took notice of instructions and responded to corrections. The impact of undesirable behaviour was reduced if the dog responded to the GDO. Dogs that did not follow commands were described by GDOs as frustrating or hard work. By contrast, some dogs were described as actively looking for instructions, which links with attentiveness.

“*She obeys orders. Spends most of the time lying there looking for instructions*.”

Their dog’s obedience gave GDOs confidence in controlling them. Some GDOs attributed this level of obedience to the bond they had with their dog and the effectiveness of their teamwork. Working in harness had an improving effect on a dog’s obedience. Some GDOs thought it was positive that their dog could differentiate between work and play, whereas others would have liked their dog to exhibit the same high level of obedience at all times.

“*Err on a lead, going to the park (he) gets excited …. He is very different. He knows he’s off duty, he’s a dog and it’s good he can differentiate*.”

#### Locating objectives

GDOs were surprised and impressed by their dogs’ ability to lead them to objects or places in the environment, such as doors, pelican crossings, shop counters or bins, even if they had not encountered that specific version of the object before. One instruction was often enough for the dog to guide them to their destination, even if they hadn’t followed the usual route or the dog had to detour around some new obstacles. When relating such behaviour, dogs were described as having the ability to solve problems, being intelligent or independent. The dog’s ability to locate objects contributed greatly to the GDO’s confidence in the dog.

“*I just love it when she finds a door, where I want to be, (it) gives a really good feeling*.”

#### Traffic

A guide dog’s behaviour in relation to traffic, which kept the GDO safe, was of huge importance to GDOs, being the single most praised issue by the GDOs when asked for an example of pleasing behaviour. Dogs may have refused to move or stopped quickly even when asked to do otherwise when there was oncoming traffic.

“*Yes on the main road to the shops I asked her to go forward twice and she refused because there was a car indicating. She stopped me from getting knocked down*”

By way of contrast some dogs were unpredictable in the face of traffic, which the GDOs attributed to being scared, distracted or close to home.

#### Calmness

GDOs often described their dogs as calm. Dogs were described as laid back, placid or even hardly noticeable when not working and around the house.

“*In home he's very good (he) sits quietly, has toys to play with, he's a perfect domestic animal*”

However, excitability did occur in situations when experiencing something new, going for a free run or meeting new or familiar people and dogs. Excitement was often linked to the immaturity of certain dogs and, although it did occur in harness, it mostly drew comment when the dog was on a lead.

“*At certain points on routes she can get over excited …but she's only newly qualified and she is quite young*.”

The physical effects of excitement were pulling on the lead and difficulty in controlling the dog, which sometimes became noisy or overactive. These behaviours caused feelings of annoyance or embarrassment in the GDOs. Dogs were sometimes described as being hard work due to excitability.

“*I wouldn’t call it disappointing, more embarrassing. (he) gets so overexcited (I) have to put him in another room to calm down, that sort of thing… from a work point of view (it’s) not really a problem, only when people come to the house*.”

#### Inappropriate non-working behaviour

Whilst many dogs were described as calm and quiet, when left, a small number displayed undesirable behaviour, including stealing, being destructive in the garden and chewing. Scavenging was discussed in a mixture of working and non-working situations. When GDOs mentioned that their dogs scavenged, they described it as opportunistic and preventing the dog from doing so centred on denying them the opportunity.

“*(He) has a thing (for) food if there's an opportunity so I don't give him the opportunity*.”

“*She can be a bit mischievous, pinch socks, thinking she can have a play. (It’s) not the end of the world. (You) think she shouldn’t be doing it (but) you have to stand back and think she’s a dog at the end of the day*.”

GDOs explained that one of the difficulties with addressing undesirable behaviour when left was that they would have to catch the dog in the act. Comparatively few GDOs made comments about their dog’s vocal behaviour, most GDOs commented positively about their dog being quiet when left. When dogs did show vocal behaviour the impact on GDOs varied, with some indicating this was annoying or embarrassing, and others saying it did not have much impact upon them.

“*People expect him to be quiet and he's not… Barking is embarrassing, (you're) drawing attention to yourself. He can be difficult then (I say) 'oh be quiet, be good*.’”

“*If someone's coming to the door he'll woof and I'm pleased with that as we have a lot of nasty people around us at the moment*.”

#### Toileting behaviour (spending)

Inappropriate toileting (defecation not in the time and space provided) caused problems for GDOs especially when their dogs wanted to defecate when working in harness. There were only two comments which related to toileting indoors. The desire to defecate also affected dogs’ working behaviour, with GDOs describing their work as ‘off’. Some GDOs were able to cope with inconsistent spending, but one described it as the worst thing for a GDO.

“*It’s one of the worst case scenarios for a blind or poorly sighted person. (it’s)embarrassing, frustrating (and) can have a negative effect on how people view guide dogs, especially if you can’t clear it up as you can’t see it*.”

“*Spending–I can deal with. I have enough vision to cope and I’ve not got issues with dog poo. I would rather not have to*.”

Coprophagia (the consumption of faecal matter) was rarely mentioned, but when it was, it was a significant problem to GDOs. One dog needed to be muzzled when free-running, whereas another’s coprophagia detracted significantly from an otherwise excellent working relationship with the dog.

#### Position and speed when in harness or on lead

The GDOs’ comments in relation to speed control mainly focused on their dog’s propensity to pull in certain circumstances, sometimes in harness, but particularly on the lead. This was often attributed to excitement as the dog knew they were not working and likely to be going for a free run and sometimes caused physical control issues for their GDOs. The importance of this was emphasised by GDOs who commented positively that their dogs would not pull in comparison with previous or other dogs.

“*He’s very good, doesn’t pull at all, walks nicely to (my) side (I) was used to one who pulled your arm off. (There’s) the odd yank for a sniff, (he’s) a pleasure to walk with on the lead*.”

By contrast a couple of GDOs mentioned that their dog would slow down in certain circumstances, which inconvenienced the GDO due to lack of attention or desire to not go home.

## Discussion

The GDOs in this study spoke a great deal about the attentiveness, obedience, confidence and consistency of their dog’s behaviour, suggesting that these were the most important aspects of their dog’s behaviour to them. It became apparent when comparing responses, that there is a clear distinction in how the GDOs see their dogs in a work versus social context. GDOs often described the attachment or bond they had to their dog and their dog’s attachment to them. In the remainder of the discussion we focus on these key results of attentiveness and obedience, confidence, consistency, differences between GDOs, handling ability and a guide dog as a working dog and pet.

### Attentiveness and obedience

Two of the most important categories for GDOs were Attentiveness and Obedience. From a GDO perspective, these could be considered key traits of a high quality guide dog. It was very important for the dogs to pay attention to their environment, their work, their GDO and, on occasion, other people. A dog that was obedient and paid attention to commands, without the need for repetition, was particularly pleasing to GDOs, creating a trusting relationship and bond between the GDO and dog. It appears especially pleasing to GDOs when the dog’s obedience does not differ between working and social situations. It is perhaps not surprising that attentiveness and obedience were of importance to GDOs as they are aspects of the dog’s behaviour that govern the dog’s interaction with the GDO. Attentiveness is also highly rated by pet dog owners (Serpell, 1983) [25]. When attentiveness and obedience were lacking, problematic characteristics of the dog such as distraction, are viewed as developing into problems that disrupt the GDO’s work and relationship with the dog. Distraction appears to be an important characteristic in qualifying as a guide dog [16,17,18, 26] and indeed this study shows that this remains an important characteristic for GDO satisfaction post qualification.

### Confidence

Dogs described as confident were considered able to guide their GDOs independently and required minimal instruction. They were also able to adapt to changes in the environment or obstacles on their routes. Confident behaviour, which kept the dog and GDO safe in the face of traffic, was the singularly most pleasing aspect of a dog’s behaviour and it can be concluded that a confident dog contributes greatly towards the confidence of the GDO. This mirrors previous findings that puppies analysed as being low in fearfulness and those that were bolder were more likely to qualify as guide dogs [15,27]. Arata et al. (2010) also found that dogs that showed more initiative were more likely to qualify as guide dogs [26].

### Consistency

Consistency of behaviour was considered a key trait by GDOs, whether this is across time or across different environments e.g. working, non-working and social situations when in harness, on a lead or neither in harness nor on a lead. Consistent behaviour is predictable, even when it is negative and this allows for the GDO to prepare themselves and their work accordingly. It is more problematic for a dog’s behaviour to be inconsistent as this leads to feelings of trepidation and uncertainty in GDOs.

Consistency of behaviour can also be linked to the clear difference in reported behaviour when in harness, on a lead or not on harness nor on a lead. Dogs’ behaviour was almost universally reported as different across these situations with dogs being in ‘work mode’ when in harness or just like a pet dog when not on harness nor on a lead. Many GDOs recognised the importance of this distinction, appreciating that dogs needed to relax when not working in harness but others found the difference in behaviour, obedience and excitability hard to manage.

### Differences between GDOs

A broader finding of this study was the importance *of the GDO* as well as the behaviour of the dog in promoting a high quality lasting partnership. GDO personality was not a focus of this study and was not formally assessed but comments from the GDOs about what they were happy or able to do to influence the behaviour of their dog were significant. The wide variety of lifestyles of individuals who own guide dogs also means that each GDO will have different requirements of their dog. This observation is consistent with previous studies. For example, Lloyd (2004) also observed the diversity of GDOs’ personalities and needs, and identified that the importance of a match of personalities between the GDO and dog was often mentioned by GDOs[28]. Personality matching between pet dogs and their owners was explored by Curb et al. (2013)[29]. In this study owners who possessed similar personality traits to their dog reported being more satisfied with their dog. Dog-owner dyads were also found to have similar personality traits in a study by Turcsán et al. (2012)[30]. The concept of personality ‘matching’ is also currently used by the American Society for the Prevention of Cruelty to Animals for people thinking of adopting a pet in an effort to ensure the animals go to an appropriate home.[31]

### Handling ability

Assessments of potential guide dogs in training are completed by experienced, professional guide dog trainers with a wide range of experience of behaviour and strategies to train and accommodate this behaviour. GDOs are less experienced dog handlers, which sometimes led to a gap between the ability of behavioural assessments of guide dogs in training to capture behaviour relevant to the GDO, sometimes masking behaviour that became problematic for the GDOs. This caused problems for the GDOs when they were not able to provide this required handling. This leads to the recommendation that behavioural profiling systems should include consideration, not only of how the dog can be successfully handled by the trainer, but whether a less experienced or skilled GDO can readily achieve the same behaviour independently when with the dog.

Previous research suggests that dog behaviour varies as a result of the handler’s behaviour[32,33,34]. These studies found that the patience of the owner was also positively associated with obedience in the dogs as well as the owners being more involved in play. Dog behaviour also seems to be affected by the attentional state of their handler[35,36,37]. In guide dogs, the ability of the GDO to control the dog was found to contribute to the compatibility between the GDO and dog[28].

The discussion of sections ‘Differences between GDOs’ and ‘Handling ability’ emphasise the role of a guide dog in partnership with its GDO, for which it needs to be matched accordingly. This may also require a greater emphasis on the compatibility, ongoing training and expectations of GDOs in addition to the training of dogs. This will help to develop and strengthen the bond between dog and GDO and increase the likelihood of successful long lasting partnerships.

### A working dog and pet

Behavioural assessments of potential guide dogs are conducted by professional guide dog trainers whose primary aim is to prepare a dog for a working role. What was emphasised by this study was that this working role, although of primary importance to the GDOs and role of the dog, often only constituted a minority of the time that the GDO was with their dog, maybe only working once or twice a day. The rest of the time the GDO was living with the dog in social situations and its behaviour in these situations was identified as of significant importance, hence our decision to use a particular quote in the title of this paper.

Surprisingly, perhaps, there is relatively little discussion of this non-work aspect of the guide-dog GDO relationship in the existing literature. Exceptions include Sanders (1999) who conducted an ethnographic study exploring the relationship between both pet and working dogs and their owners/handlers, including guide dogs and their visually impaired GDOs[8]. Furthermore, Michalko (1999) published an account of his experiences of being a GDO and his relationship with his dog[38]. Both of these detailed accounts describe the strength of the bond formed between the dog and GDO and how the relationship revolves around communication, trust and interdependence. In summary, guiding work is only one aspect of a complex relationship and this needs to be taken into account when matching dogs and GDOs.

### Limitations

As acknowledged the chosen methodology of this project has limitations. For example, the responses of the GDOs were recorded by the researcher taking notes, rather than audio recorded, during the conversation with the GDO on the telephone. The potential for omission of comments and misunderstanding of responses is, therefore, potentially higher. Effort was made, however, to make notes as full and verbatim as possible. On balance it was felt that recording conversations may have been experienced as intrusive by respondents affecting the intended conversational account of their dog’s behaviour intended by the research

It is also worthy of note that only one researcher (PC) carried out the telephone survey and took primary responsibility for data analysis. Whilst this gives the research a consistency of approach and perspective it needs to be noted that this research is subject to the systematic, conscious or unconscious bias associated with an individual. This was mitigated throughout the research process by discussion of findings and consultation with other team members and experts from Guide Dogs more widely.

Certain aspects of the survey population needs to be taken into account. First of all they were drawn from a list of GDOs who had indicated that they would be prepared to be contacted regarding participation in research projects. This means that the pool of potential participant GDOs did not represent the entire population of GDOs, and may, potentially, have excluded those who felt less positive about their guide dog experience.

Finally, for individuals to be eligible for the study they were required to currently have a guide dog. The GDO was then asked to respond to the questions in relation to their current dog. This means that the comments, findings and conclusions from this study all relate to currently successful guide dog and GDO partnerships, even though some of the partnerships were very new at just 5 days old. No conclusions could therefore be drawn about the type of behaviour that may lead to the breakdown of a partnership.

## Conclusion

When surveyed, safe behaviour in the face of traffic was the most important positive aspect of a guide dog’s behaviour. The most significant negative aspect was pulling or high tension on the lead and /or harness, which reduces the satisfaction of guide dog ownership. Other aspects of guide dog behaviour were highlighted as particularly pleasing or disappointing by GDOs. Many of these behaviours are already captured in behavioural assessments by professional guide dog trainers during training.

However, a number of important areas of behaviour were revealed that were not considered in behavioural assessments for dogs during guide dog training and consideration of these elements would potentially enhance these assessments. Key areas were; consistency of behaviour, the dog’s maturity and the dog’s behaviour in relation to children. Social behaviour was also highlighted as being of great importance to GDOs. Many comments showed that how their dog behaved when not working in harness had a significant positive or negative effect on the GDO and their relationship with the dog. Aside from specific guiding tasks, the findings of this study fit with those found in the pet owning population, suggesting that views on appropriate and inappropriate behaviour may apply to both pet and working dogs.

Finally, the survey revealed the breadth in what GDOs considered problematic or pleasing. This finding confirms the importance of matching; there may be no one size fits all approach when considering the ideal guide dog. Some GDOs found behaviour that was problematic to others pleasing and some found potentially problematic behaviour endearing. Further research is needed to understand which qualities of dogs and owners interact to form successful partnerships in both pet and working dogs and how the expectations of owners can be managed to ensure this.
